# Case Report: A testicular torsion as an initial presentation of a patient with metastatic cecum signet-ring cell cancer

**DOI:** 10.3389/fonc.2023.1189552

**Published:** 2023-09-08

**Authors:** Bo Yu, Mi Meng, Rui Qin, Qiang Xu, Neng Zhang, Ni Fu

**Affiliations:** ^1^ Department of Urology, The Second Affiliated Hospital of Zunyi Medical University, Zunyi, China; ^2^ Department of Oncology, The Second Affiliated Hospital of Zunyi Medical University, Zunyi, China; ^3^ Department of Urology, The Affiliated Hospital of Zunyi Medical University, Zunyi, China

**Keywords:** colon cancer, signet ring cell carcinoma, testicular metastases, case report, pathological features

## Abstract

Secondary neoplasms of the testes from solid tumors are rare and usually present as a painless mass. Metastatic cecum signet-ring cell cancer of the testis is extremely rare. The orchioncus usually shows hypervascularity on color Doppler ultrasound. The present study reports an unusual case of testicular secondary signet-ring cell carcinoma mimicking missed testicular torsion in a 55-year-old male patient with right scrotal swelling and intermittent pain for 10 days. As color Doppler ultrasound showed an avascular distribution of the enlarged right testis, missed testicular torsion was initially diagnosed. Right-sided orchiectomy was performed, and pathology of the resected testis revealed an intestinal-type adenocarcinoma with mucinous and signet-ring cell features. This pathological feature led to further endoscopic colorectal biopsy of the digestive tract, which revealed poorly differentiated adenocarcinoma of the cecum with signet ring cell features similar to those of testicular specimens. In conclusion, differential diagnosis should be considered for rare testicular neoplasms, as was seen in this rare occurrence of testicular torsion in a patient who initially presented with metastatic colorectal cancer. A correct preoperative diagnosis can change the management and outcome. This report shares our reasons for misdiagnosis and opinions on diagnosing and treating this kind of case.

## Introduction

Colorectal signet-ring cell carcinoma is a subtype of colorectal mucinous adenocarcinoma and is a rare and aggressive disease, accounting for only 1% of colorectal carcinomas (1). Signet ring cell carcinoma (SRCC) was first proposed by Saphir and Laufman in 1951; SRCC is composed of at least 50% of neoplastic cells showing signet ring cell (SRC) morphology. Tumor cells producing intracellular mucin that displaces the nucleus to the periphery characterize SRCC (2). Patients with early-stage colorectal signet-ring cell carcinoma cancer are often asymptomatic, and approximately 30% to 40% of patients present with distant metastases at the time of diagnosis. The most common sites of metastasis are the liver, peritoneum, and lymph nodes. In more advanced stages, colorectal SRCC cancer is still often asymptomatic, and many patients present with metastases in the lungs, brain, bones, soft tissues, and other sites (3). In men, it is rare for colorectal SRCC cancer to metastasize to the testes. Only a few cases of testicular metastasis from colorectal cancer have been reported in the literature (4).

In rare instances, testicular swelling can be an initial presentation of metastatic cancer. The present study reported a case of a man who initially presented with right testicular swelling pain and was eventually diagnosed with secondary testicular cancer from an aggressive metastatic colorectal signet-ring adenocarcinoma.

## Case presentation

**Figure 1:**
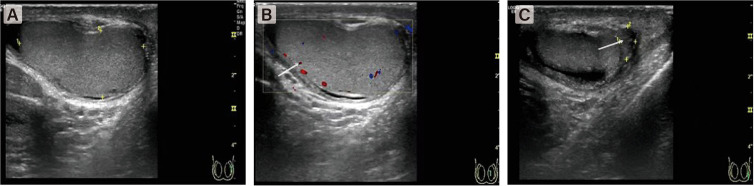
Color Doppler US image shows a few of color flow signal at the intratesticular tissue. **(A)** The right testicle was enlarged and axial position changed. **(B)** The blood flow signal in the testis was significantly reduced. **(C)** The boundary between epididymis and testis is not clear.

**Figure 2:**
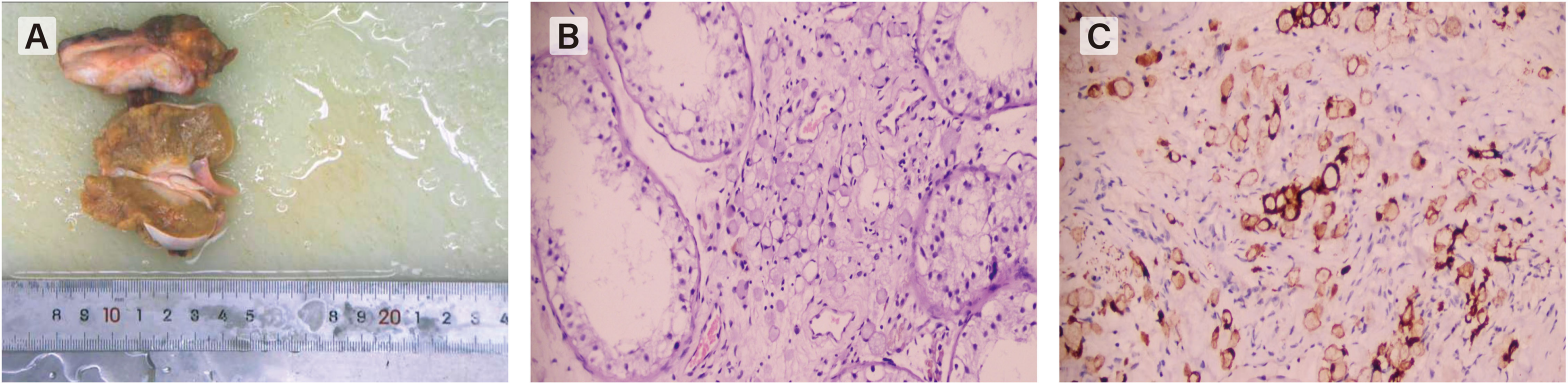
**(A)** The gross specimen shows a diffusely enlarged testis which have an unclear boundary from the epididymis. **(B)** Hematoxylin-eosin staining(HE×200) shows signet-ring cells characterized by large cytoplasmic vacuoles indenting the nucleus, appearing either empty clear or containing a droplet of mucin. **(C)** positivity by immunohistochemistry showed positive expression of Villin-2 proteinconfirmed the epithelial and gastrointestinal origin.

**Figure 3:**
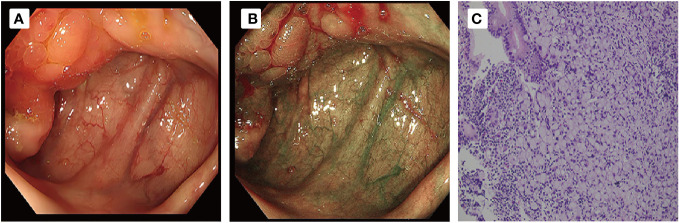
**(A, B)** Colonoscopy and examination showed that the ileocecal mucous membrane was edema and ero- sion, with nodular surface changes and unclear boundary involving the opening of the appendix. **(C)** Pathological examination ((HE,×20)) revealed signet ring cell carcinoma of ileocecal part.

**Figure 4:**
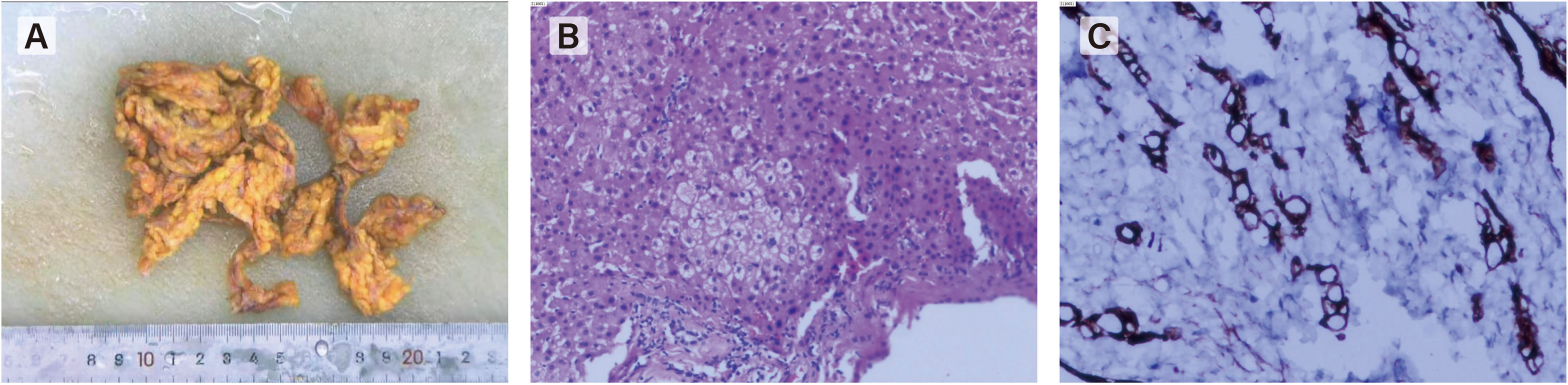
Laparoscopic exploration revealed a large number of metastatic nodules in peritoneum, greatero- mentum, small intestine and liver. **(A)** gross specimen of greateromentum. **(B, C)** Histopathological examination of the omentum revealed met- astatic signet ring cell carcinoma.

A 55-year-old male patient presented to the emergency department of our hospital due to swelling of the right testicle for 10 days and worsening for 1 day. Physical examination indicates axial changes in the testicles, accompanied by swelling and hardening of the testicle. The patient has no symptoms such as abdominal pain, bloating, or diarrhea, no family history of intestinal tumors, and no abnormalities found on digital rectal examination. Testicular ischemia in the enlarged right scrotum was observed by color Doppler ultrasound (**Figure 1**). Acute testicular torsion was suspected. The patient underwent a right-sided orchidectomy after scrotal exploration. Postoperatively, the tumor of the right testis was diagnosed as intestinal-type adenocarcinoma with mucinous and signet-ring cell features by pathologists (**Figure 2**). The characteristic pathological results led to further investigations for a gastrointestinal (GI) primary cancer. No positive findings were found by upper gastrointestinal endoscopy. A staging fluorine-18 fluorodeoxyglucose positron emission tomography scan also unrevealed extensive peritoneal nodules and lesions of the digestive system. A colon endoscopy revealed a 2-cm fungating mass at the junction of the ileum and cecum of the digestive system that extended along the cecum. The pathology of the biopsy revealed poorly differentiated adenocarcinoma with signet-ring cell features similar to those in the excised testicular specimen (**Figure 3**). The patient underwent an exploratory laparoscopic approach that revealed extensive peritoneal metastasis of the tumor (**Figure 4**). He refused palliative chemotherapy with 5-fluorouracil and oxaliplatin and eventually opted for traditional Chinese medicine treatment. Finally, the patient has a survival time of one year and six months.

## Discussion

Primary testicular tumors account for over 90% of testicular neoplasms; pathologically, they are mainly germ-cell tumors that originate from the testes (5). Secondary neoplasms of the testes from solid tumors are rare in the clinic. Most of them are found incidentally by autopsy or testicular surgery for other reasons (6). In a series of 738 adult male autopsies, Garcia-Gonzalez found that patients with secondary testicular tumors were generally >50 years old, with a mean age of 60 years. Among testicular metastases, the primary solid tumors of origin are derived mainly from the prostate and lung and rarely from the colon (7). The primary tumor was derived from testicular metastasis of a colon lesion, and the pathological type was basically adenocarcinoma (5). We reviewed the literature and found that metastatic lesions of colorectal signet ring cell carcinoma and adenocarcinoma involve the breast, myometrium iris, cervix, and testis (4, 8–11). There are no reports of testis metastasis of signet ring cell carcinoma in the cecum.

According to the different origin sites, the locations of digestive tract SRCC are divided into gastric signet-ring cell carcinoma and colorectal SRCC. The most common location of primary signet-ring cell carcinomas is the stomach (1). Colorectal signet-ring cell carcinoma is extremely rare among all patients with colorectal cancer. Its characteristics include more advanced stages and younger age at presentation, lower curative resection rate, higher local lymphatic spread and peritoneal metastasis, aggressive biological behavior and poorer prognosis than ordinary colorectal adenocarcinoma (2, 3). Although the prognosis of advanced Signet ring cell carcinoma is poor, studies have shown that SRCC in colorectal cancer patients can benefit from chemotherapy, Targeted therapy and immunotherapy. Because colorectal cancer SRCC is a heterogeneous subgroup with different potential molecular mechanisms, individualized treatment based on the molecular characteristics of different patients is important. For example, the use of Topoisomerase inhibitors may have a better effect on tumors that lack the expression of SMAD4. For SRCC with PD-L1+hypermethylation, ICI therapy may be a potential effective treatment method. Considering that the median survival time of advanced Signet ring cell carcinoma is about 20 months, traditional Chinese medicine treatment did not bring obvious benefits to this patient. The most common metastatic site is peritoneal seeding. There is a low incidence in the liver, lung and bone, with uncommon metastatic sites also in the vertebrae, extraocular muscles, bone marrow and skin (5–7). The acknowledged symptom of testicular secondary neoplasms is usually different from that of testicular torsion, in which the former presents as a growing painless mass over weeks or months after the history of other tumors and the latter presents as sudden severe scrotal pain over several hours. Our report shows a rare occurrence of testicular torsion with an initial presentation of aggressive metastatic cecum signet ring cell carcinoma.

Acute testicular pain in all prepubertal and young adult males should be considered testicular torsion until proven otherwise. When a patient presents with testicular swelling and sudden severe scrotal pain, testicular torsion should be suspected based on the clinical examination and ultrasonographic examination of the testes. Testicular torsion accounts for 13%-15% of acute scrotal diseases in children, and occurs annually in 1 in 4000 men under 25 years of age (12). Testicular torsion is most likely diagnosed when color Doppler ultrasound shows a loss of blood flow in the testicles in men with testicular pain. It has been reported that color Doppler US has a quite high sensitivity and specificity in adults (13).

In this case, the patient complained about scrotal swelling with scrotal pain for 10 days. This patient underwent a scrotal exploratory orchiectomy because the initial misdiagnosis was acute testicular torsion due to the absence of vascularity on a color Doppler ultrasound. In 2017, Alrabeeah et al. reported a case of testicular seminoma that presented with acute pain and was misdiagnosed as testicular torsion (14). He considered the reason that led to the masking of the diagnosis, in that case, was the inability to properly assess the testis on physical examination, which was caused by the surrounding scrotal edema, as well as the nonspecific changes on ultrasound imaging, which led to a provisional diagnosis of testicular torsion. This ultimately led to a defect in management of the case. Nevertheless, the causes of sudden scrotal pain in testicular cancer patients have been described in the previous literature and were mostly attributed to the tumor itself. It was noted that in such cases, both intratesticular bleeding within the tumor itself and even torsion due to the mass effect of the tumor caused testicular cancer to present as acute pain in the scrotum (15). Hence, the high index of suspicion for testicular torsion should always be the priority when a patient presents with sudden onset testicular pain, but testicular neoplasms should never be overlooked within the differential diagnosis. Proper physical examination and additional imaging, such as CT and MRI, play an important role in the differential diagnosis of testicular enlargement for correct preoperative diagnosis. This is significant as it alters both the surgical approach; orchidectomy must be performed and that orchidopexy should be chosen to salvage the testis in an isolated testicular torsion case.

This patient underwent scrotal exploration orchiectomy for acute testicular torsion, and postoperative incidental pathology confirmed testicular metastasis of aggressive cecum signet ring cell carcinoma. Metastatic spread of a solid tumor to a testicular site is rare, which often indicates widespread metastatic disease and has a poor prognosis. The material cause of the low incidence of secondary testicular tumors is that the testes are protected by the distinct anatomy that is offered by the blood-testis barrier. The other reason for the low incidence of secondary testicular tumors is the tunica vaginalis as an external fibrous covering sheath of the testis, which separates the testis from the peritoneal cavity and provides additional protection.

There are several hypotheses for the route of tumor metastasis to the testis: 1. hematogenous spread; 2. retrograde venous spread or embolization, such as renal and adrenal tumors along the internal spermatic vein; 3. retrograde lymphatic spread; 4. spread along the vasdeferens or sphincter, such as prostate cancer and intra-abdominal carcinoma; and 5. direct infiltration, such as malignant tumors of the epididymis and spermatic cord. The exact mechanism of spread of digestive tract signet ring cell carcinoma is unknown, but many theories have suggested that the routes of peritoneal seeding play a dominant role in secondary metastatic spread. Among colorectal cancers, the diffuse colorectal cancer subtype is commonly associated with signet-ring cells. These cells lack adhesion and invade as single cells or small groups, leading to scattered tumor cells and a higher probability of seeding (16). Since most cases of testicular metastases present as hydrocele and peritoneal carcinomatosis, it is proposed that there may be microscopic channels of communication between the peritoneum and testes. Our patient exhibited extensive peritoneal carcinomatosis, so peritoneal seeding likely played a dominant role in disseminating the cancer to the testis.

When signet ring cells are encountered in an orchiectomy specimen, it is not easy to identify a primary extratesticular site of origin since the possible primary sites of signet ring cell metastatic adenocarcinomas are broad and may include the stomach and other GI and pancreaticobiliary sites, as well as the lung, bladder, breast, and gynecologic tract. Multiple means of examination can be used to exclude the differential diagnosis and to search for the primary tumor. Immunohistochemistry staining often plays an important role in identifying the source of metastatic cancer cells. CK20(+) and CK7 (–) are suggestive of a colorectal origin, thyroid transcription factor (TTF)-1 (+) is suggestive of a lung origin, and GCDFP-15(+) is suggestive of a breast origin. Notably, signet ring cell-like changes are also seen in some nonneoplastic lesions, such as pseudomemilous colitis, cystic fibrosis, and ulcerative colitis, and can also result from cytosolic contraction after formalin fixation. Therefore, immunohistochemistry is of limited value in establishing the primary site. The diagnosis for stage and grade of tumor still needs to be determined based on the clinical basis, as was done in our patient. Since a majority of signet ring cell tumors originate in the stomach and colon, when we accidentally find metastatic signet ring cell carcinoma after testicular surgery, further CT, gastroenteroscopy and staging fluorine-18 fluorodeoxyglucose positron emission tomography scans are necessary to find the primary site and evaluate the TNM stage of the tumor. The National Comprehensive Cancer Network has published the current guidelines related to stage IV colon cancer, and they recommend that resectable primary lesions and metastatic lesions should be removed together and given postoperative systemic chemoradiation. Therefore, the patient underwent an exploratory laparoscopic approach on account of only a 2-cm fungating mass revealed by colon endoscopy, and 18F-FDG PET/CT revealed peritoneal nodules. Unfortunately, the exploratory laparoscopic approach revealed extensive peritoneal metastasis of the tumor in this case. This case also indicated that a staging Fluorine-18 fluorodeoxyglucose positron emission tomography scan has unique clinical application value in the diagnosis and staging of mixed-SRC and non-SRC, but the diagnosis and staging of SRC have limitations (17), as it needs to be combined with other tests to make a comprehensive judgment.

When signet-ring cells are encountered in an orchiectomy specimen and the primary extratesticular site of origin cannot be determined, it is important to consider primary testicular tumors in the differential diagnosis. Many primary testicular tumors, such as primary testicular signet ring cell stromal tumor (PSRST), signet ring cell dominant seminoma and primary signet ring cell germ cell carcinoma of testes (PSRGCT), have a signet ring morphology. PSRST is a benign tumor with good prognosis. Spermatogonia variants with signet ring cell dominance and PSRGCT are considered germ cell tumors and are also considered to have a good prognosis. The treatment of choice for the pure form of testicular solid tumor without extra testicular metastasis is radical orchiectomy, and no further postoperative therapy is considered necessary (18).

Because differentiation of metastatic testicular tumor from a primary testicular tumor has significant implications for management and survival, diagnosis of primary testicular tumor should only be made when metastasis is excluded by mandatory extensive examination of the tumor.

We emphasize that when such patients are encountered, proper physical examination and additional imaging, such as CT and MRI, would be helpful for correct preoperative diagnosis. For metastatic tumors of the testis found incidentally after surgery, the combined application of immunohistochemistry, endoscopy and PET-CT can help to locate the primary lesions.

## Conclusions

In conclusion, when an enlarged testis demonstrates an absent blood signal on color Doppler US in patients, not only testicular torsion but also testicular tumors should be considered for differential diagnosis. Proper physical examination and additional imaging, such as CT and MRI, play an important role in the differential diagnosis of testicular enlargement. Meanwhile, immunohistochemistry, endoscopy and PET-CT can be used to locate the primary lesions for metastatic tumors of the testis.

## Data availability statement

The raw data supporting the conclusions of this article will be made available by the authors, without undue reservation.

## Ethics statement

Written informed consent was obtained from the individual(s) for the publication of any potentially identifiable images or data included in this article.

## Author contributions

BY and QX were the patient’s Urologists, reviewed the literature and contributed to manuscript drafting. MM, RQ were responsible for the revision of the manuscript for important intellectual content. All authors read and approved the final manuscript.
